# Supplementary material for: A Glimpse into the Structural Properties of the Intermediate and Transition State in the Folding of Bromodomain 2 Domain 2 by Φ Value Analysis
Source: Int J Mol Sci. 2021 May 31;22(11):5953. doi: 10.3390/ijms22115953 (PMC8199192; doi:10.3390/ijms22115953)
Supplement: Supplementary file 1 [file ijms-22-05953-s001.zip › ijms-1227644-supplementary.pdf]

A glimpse into the structural properties of the intermediate and transition state in the folding of Bromodomain 2 domain 2 by Φ-value analysis.

Leonore Novak [1†], Maria Petrosino [1†], Daniele Santorelli [1†], Roberta Chiaraluce[1], Valerio Consalvi [1], Alessandra Pasquo [2] and Carlo Travaglini-Allocatelli[1,*]

[1] Dipartimento di Scienze Biochimiche "A. Rossi Fanelli", Sapienza University of Rome, Roma (Italy);leonore.novak@uniroma1.it (L.N.), maria.petrosino@uniroma1.it (M.P.),daniele.santorelli@uniroma1.it (D.S.),roberta.chiaraluce@uniroma1.it (R.C.), valerio.consalvi@uniroma1.it (V.C.), carlo.travaglini@uniroma1.it (C.T.)

[2] ENEA CR Frascati, Diagnostics and MetrologyLaboratory FSN-TECFIS-DIM Frascati RM (Italy); alessandra.pasquo@enea.it

[†] These authors contributed equally to this work

[*] Correspondence: carlo.travaglini@uniroma1.it

a

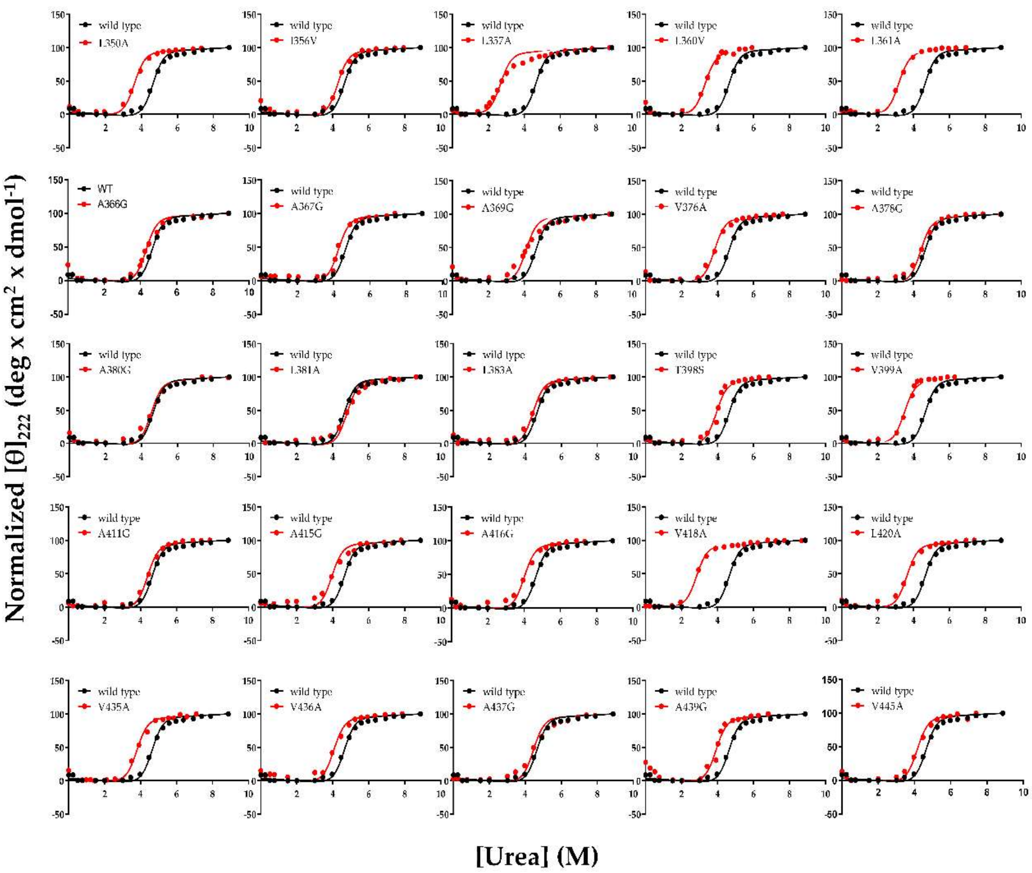

b

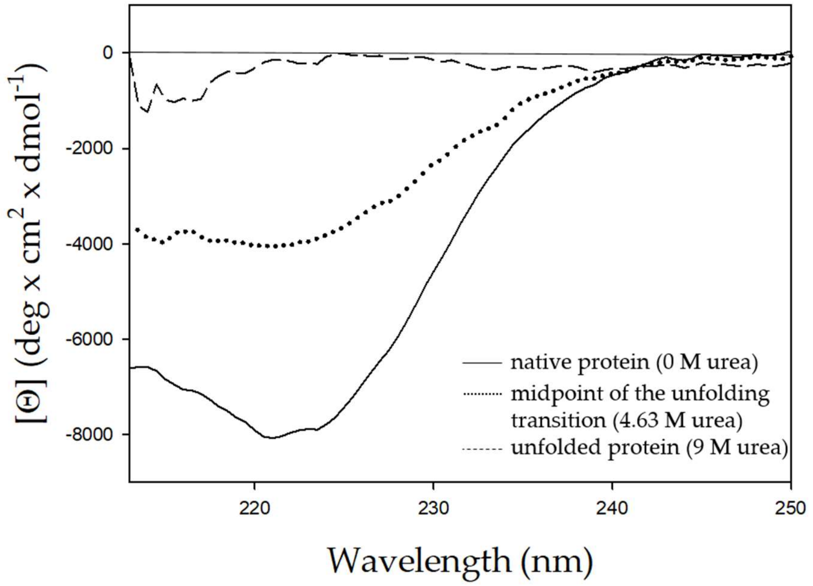

Figure S1. Urea-induced equilibrium unfolding of BRD2(2) wild type and mutants. (a) Plot of the molar ellipticity at 222 nm as a function of denaturant concentration of BRD2(2) wild type and mutants. The continuous lines represent the nonlinear global fitting of the molar ellipticities at 222 nm data ($[\theta]_{222}$) calculated as described in Materials and Methods. Data were normalized between 0 and 100%, where 0 corresponds to the molar ellipticity at 222 nm of the native protein, the smallest value, (at 0 M urea), and 100 corresponds to the molar ellipticity at 222 nm of the unfolded protein, the largest value, (at 9 M urea). Normalization was calculated using GraphPad Prism 5 as follows: $z_i = (x_i - \min(x)) / (\max(x) - \min(x)) * 100$, where z_i is the i_{th} normalized value in the dataset, x_i is the i_{th} value in the dataset, min(x) is the smallest value in the dataset and max(x) is the largest value in the dataset. (b) Far-UV CD spectra of BRD2(2) wild type. The continuous line represents the far-UV CD spectrum of native protein (0 M urea), the dotted line represents the far-UV CD spectrum at the midpoint of the unfolding transition (4.63 M urea) and the medium dashed line represents the far-UV CD spectrum of the unfolded protein (9 M urea). Spectra were recorded between 213 and 250 nm.

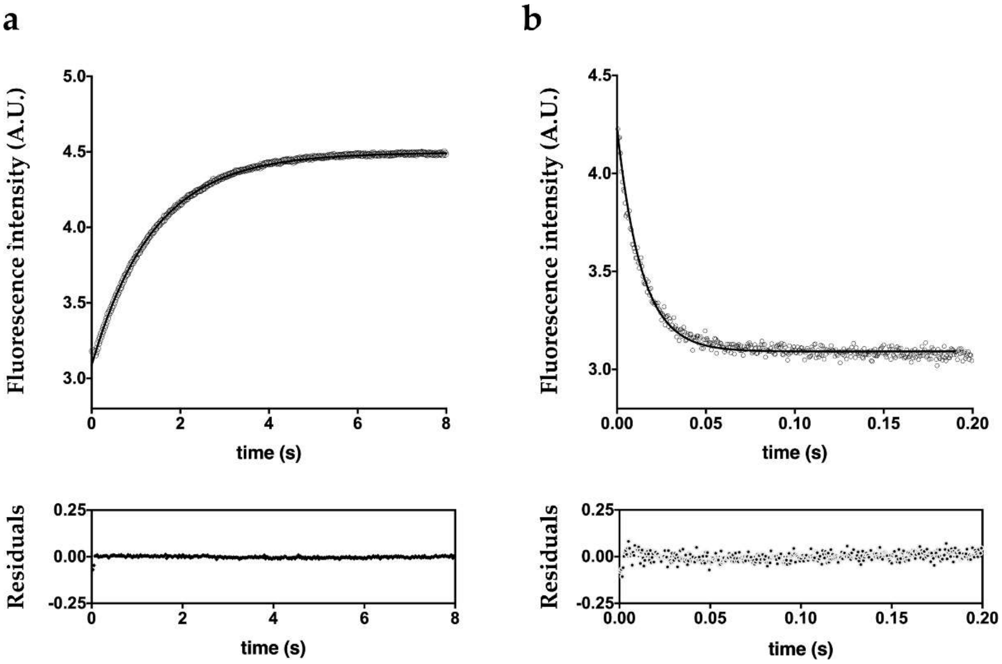

Figure S2. Representative kinetic traces of BRD2(2) A367G mutant for unfolding (a) ([Urea]=8.1 M) and refolding (b) ([Urea]=0.7 M) together with their relative residuals plot. The line represents the best fit to a single exponential decay model ($Y = a*e^{(-k*t)} + c$) without any restrain applied: the parameter obtained for unfolding are $k = 0.72 \pm 0.001\ s^{-1}$ and $a = 1.41 \pm 0.001$, while for refolding are $k = 72.5 \pm 1.7\ s^{-1}$ and $a = 1.1 \pm 0.01$. The goodness of fit (R square) is 0.9998 and 0.9979 for unfolding and refolding traces respectively.
